# Genome Sequence of Lymphocystis Disease Virus 2 LCDV-JP_Oita_2018, Isolated from a Diseased Japanese Flounder (Paralichthys olivaceus) in Japan

**DOI:** 10.1128/MRA.00547-21

**Published:** 2021-08-19

**Authors:** Satoshi Kawato, Reiko Nozaki, Ikuo Hirono, Hidehiro Kondo

**Affiliations:** a Laboratory of Genome Science, Tokyo University of Marine Science and Technology, Tokyo, Japan; Queens College CUNY

## Abstract

Here, we present the genome sequence of lymphocystis disease virus 2 LCDV-JP_Oita_2018 (genus *Lymphocystivirus*, family *Iridoviridae*), which was isolated from a diseased Japanese flounder (Paralichthys olivaceus) in Japan. The LCDV-JP_Oita_2018 genome was assembled into a circular contig of 186,627 bp, with 140 predicted protein-coding genes and a GC content of 27%.

## ANNOUNCEMENT

Lymphocystis disease (LCD), which is caused by LCD virus (LCDV) (genus *Lymphocystivirus*, family *Iridoviridae*), is a common viral disease in fish (1–6). Here, we sequenced the genome of LCDV 2 LCDV-JP_Oita_2018, which was isolated from a Japanese flounder (Paralichthys olivaceus).

A dead P. olivaceus fish exhibiting tumor-like lesions on the body surface (a typical clinical sign of LCD) was provided by a commercial farm in Oita Prefecture, Japan, in 2018. Skin lesions were excised, homogenized in phosphate-buffered saline, and stored at −30°C. Two milliliters of thawed lysate was diluted with 6 ml of TNES-urea buffer (6 M urea, 10 mM Tris-HCl [pH 7.5], 125 mM NaCl, 10 mM EDTA, 1% SDS) (7) and treated with proteinase K (final concentration, 500 ng/ml) for 2 h at 60°C. Five milliliters of 5 M NaCl was added, and the lysate was cleared by centrifugation. Genomic DNA was extracted from the supernatant using the cetyltrimethylammonium bromide (CTAB) method (8). The crude DNA was purified using a NucleoBond AXG 100 column (Macherey-Nagel). No shearing or size selection of the extracted DNA was performed before library preparation. Default parameters were used for all software unless otherwise noted.

A paired-end library was prepared with the Nextera XT library preparation kit (Illumina) and was sequenced with the MiSeq reagent kit v2 (2 × 150 bp) (Table 1). The raw Illumina reads were quality trimmed using Fastp v0.20.0 (9) and were *de novo* assembled by SPAdes v3.13.0 (10), to generate a draft LCDV-JP_Oita_2018 genome.

**TABLE 1:** LCDV-JP_Oita_2018 sequencing statistics

Parameter	Data for:			
	Illumina reads (accession no. DRR213899)		Nanopore reads (accession no. DRR213900)	
	Raw	Filtered	Raw	Filtered
No. of reads	3,682,911	3,659,510	97,124	1,065
Total length (bp)	867,436,261	863,771,560	630,612,147	71,205,129
Avg read length (bp)	118	118	6,493	66,859
*N*_50_ (bp)			15,501	64,930
No. of mapped reads	2,570,459	2,530,934	66,333	1,065
Proportion mapped (%)	69.79	69.16	68.30	100.00
Mean coverage^*a*^ (×)	3,137	3,161	2,480	372

a Calculated using SAMtools coverage v1.10 (12) from alignments generated by minimap2 v2.17 (11), with the settings ax sr for Illumina reads and ax map-ont for Nanopore reads.

A long-read library was prepared with a ligation sequencing kit (SQK-LSK109; Oxford Nanopore Technologies) and was sequenced using an R9.4.1 flow cell on a GridION platform (Table 1). The fast5 files were base called using Guppy v3.2.8+bd67289 with the fast mode.

The Nanopore reads were mapped to the draft LCDV-JP_Oita_2018 genome using minimap2 v2.17-r963-dirty (11). The mapped Nanopore reads were extracted using SAMtools v1.10 (12) and filtered by length (>50 kb) using SeqKit v0.10.0 (13). The filtered Nanopore reads (Table 1) were *de novo* assembled using Flye v2.6 (14), yielding a single contig. The filtered Nanopore reads and trimmed Illumina reads were mapped back using minimap2 to prepare input for polishing with HyPo v1.0.2 (15). Protein-coding genes were predicted using Prodigal v2.6.3 (16). The proteins were manually annotated based on BLASTP search results with the NCBI nonredundant protein database (accessed February 2020).

The LCDV-JP_Oita_2018 genome was assembled into a 186,627-bp circular sequence with a GC content of 27% and 140 predicted protein-coding genes. BLASTN pairwise alignment (task megablast v2.11.0+) with a Chinese isolate (LCDV-C, 186,250 bp [GenBank accession number AY380826]) revealed 99.91% nucleotide identity with 99% coverage. The small amount of divergence between the two LCDV isolates supports the view that LCDV strains affecting P. olivaceus in East Asia are virologically identical, as suggested by the high nucleotide identities (≥99.6%) of major capsid protein gene sequences (5).

### Data availability.

The LCDV-JP_Oita_2018 genome is available in DDBJ/EMBL/GenBank with the accession number LC534415. The raw read data are available with the accession numbers DRR213899 and DRR213900.
